# The Effect of Sorbent Composition on Sorption Properties of Materials Based on Ti-Ca-Mg Phosphates

**DOI:** 10.3390/ijms24097903

**Published:** 2023-04-26

**Authors:** Natalia Mudruk, Marina Maslova

**Affiliations:** Tananaev Institute of Chemistry—Subdivision of the Federal Research Centre “Kola Science Centre of the Russian Academy of Sciences” (ICT KSC RAS), 184209 Apatity, Russia

**Keywords:** synthesis, titanium phosphates, calcium phosphates, magnesium phosphates, sorption, sorbent, radionuclides, toxic metal ions

## Abstract

Individual titanium and calcium–magnesium phosphates are widely known as effective sorbents. The sorption processes on these phosphates are based on different mechanisms. The sorption efficiency towards different cations depends on the phase composition of the sorbent. Composite materials with various ratio Ti:(Ca+Mg) have been synthesized. The sorption properties of samples obtained towards Cs^+^, Sr^2+^, Co^2+^, Cd^2+^, Zn^2+^, Cu^2+^, and Pb^2+^ have been studied to establish the effect of sorbent composition on metal removal. The adsorption isotherms have been analyzed using the Langmuir, Freundlich, and Redlich–Peterson models. The composition of sorbents has no effect on the level of removal of readily hydrolyzable Pb^2+^ and Cu^2+^ cations. Removal of lead occurs preferentially via the precipitation of metal phosphates and hydroxides. Copper precipitates as hydroxide in case of a high share of Ca-Mg phosphates in the composite sorbent. The removal of cesium proceeds according to the ion exchange mechanism only. For Cd^2+^, Co^2+^, Sr^2+^, and Zn^2+^ cations, the sorption efficiency on the composite materials synthesized is found to increase with the increase in titanium phosphate’s share in the sample. All composite sorbents synthesized demonstrated a considerable increase in the level of purification of solutions studied compared with individual Ti and Ca-Mg phosphates due to the synergism of the components.

## 1. Introduction

Manmade environmental pollution leads to the accumulation of heavy metal cations in natural waters, which adversely affects the quality of water, food, and human health [1,2]. Long-term exposure of human organisms to heavy metals leads to damage to mental function and the central nervous system, blood composition, lungs, liver, and other vital organs [3]. Exceeding the maximum allowable concentrations (MAC) of Co, Cu, Zn, and Pb cations in water bodies used by humans leads to a negative effect on public health [4]. In 2011, after the accident at the Fukushima nuclear power plant (Japan), a significant amount of radioisotopes such as ^90^Sr, ^134^Cs, and ^137^Cs were released into the environment, and seawater, groundwater, the land surface were polluted [5]. The increased content of strontium in the environment results in a gradual substitution of calcium by strontium in bones which poses a serious risk to health. Both metallurgy activity and insufficient degree of wastewater treatment lead to pollution of natural water bodies [6].

The choice of the optimal method and material for the purification of solutions with certain kinds of impurities is a challenge for researchers worldwide. One of the most important tasks of applied chemistry currently is the development of purification methods for discharges from toxic metals and radionuclides.

There are widely known methods of removal of heavy metal cations from aqueous solutions based on the processes of chemical and electrochemical deposition [7], coagulation [8], flotation [9], reverse osmosis [10], extraction [11], adsorption [12,13], and their various combinations [14,15,16]. For the purification of effluents, a variety of materials are used—from natural mineral and organic substances [17,18,19,20], processed wastewater from industries [21], to composite organic–inorganic compounds obtained with a complex and costly multi-step synthesis [22,23,24].

At present, in the field of synthesis of new sorption materials, the efforts of researchers are mainly focused on obtaining hybrid (organic–inorganic) materials [25,26,27]. The synthesis of such materials is usually a complex and multi-step process that limits their application in industry. Composite inorganic sorbents have an advantage over hybrid ones, as the former can form mineral-like compounds capable of binding toxic metals, including radionuclides. Additionally, inorganic compounds are well-compatible with matrices for radionuclide disposal due to the absence of radiolysis in the inorganic matrix. The degree of binding of ions with sorbent matrix is an important parameter for metal removal, as heavy metals have very low MAC. From the standpoint of secondary pollution risk, due to the slight desorption of metals, phosphate-based sorbents are of great interest. They are capable of forming insoluble heavy metal phosphates or strongly bound metal phosphates in the sorbent matrix that prevent any desorption. Among phosphorus-containing sorbents, titanium phosphates and calcium–magnesium phosphates of various compositions are widely known. These compounds can be obtained by chemical precipitation [28,29,30,31,32]. The sorption proceeds according to the ion exchange mechanism for titanium phosphates. Purification of solutions from metal cations occurs according to the conversion mechanism for calcium and magnesium phosphates due to the difference in the solubility of calcium and magnesium phosphate compounds and toxic metal phosphates. Since sorbents based on calcium and magnesium phosphates cannot be regenerated, it is important to use low-cost mineral raw materials or industrial wastes as precursors.

Previously [33], our group reported on the synthesis of cost-effective composite inorganic sorbent based on titanium, calcium, and magnesium phosphates (TiCaMgP). The initial precursors were dolomite and titanium-containing salt, which is an intermediate product of local apatite-nepheline ore processing [34]. The final product with molar ratio Ti:(Ca+Mg) of 1:3.7 had the following phase composition: TiO(OH)H_2_PO_4_·H_2_O, Ti(HPO_4_)_2_·H_2_O, CaHPO_4_·2H_2_O, MgHPO_4_·3H_2_O, NH_4_MgPO_4_·6H_2_O. This composite material is a highly efficient sorbent toward Sr, Cs, Co radionuclides and toxic metal cations (Co, Zn, Cd, Pb) due to the synergism of individual components of material [35,36]. It is reasonable to assume that the phase ratio in the final product will determine sorption efficiency towards different cations because the sorption process is based on two different mechanisms. Therefore, the purpose of this work was to determine the effect of the composition of composite sorbents based on Ti, Ca, and Mg phosphates on the sorption properties toward Cs, Sr, Co, Cd, Zn, Cu, and Pb cations. The obtained data allow for establishing the flexibility of the proposed synthesis method, where the composition of the sorbent can be varied to purify solutions of target composition.

## 2. Results and Discussion

### 2.1. Characterization

**Figure 1:**
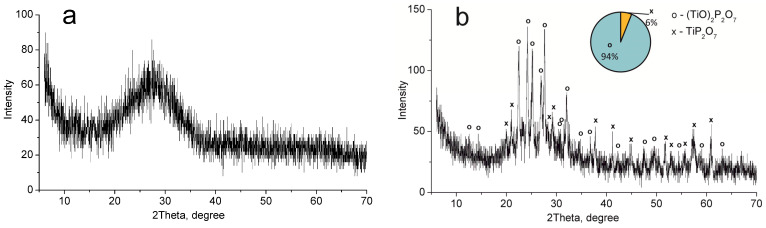
XRD patterns of the individual phases of titanium phosphates synthesized (TP sample): (**a**) 60 °C, (**b**) 850 °C.

XRD analysis showed that titanium phosphate synthesized (TP) has an amorphous structure (Figure 1a). The mixture of (TiO)_2_P_2_O_7_ and TiP_2_O_7_ phases was detected after the sample calcination at 850 °C (Figure 1b). These phases correspond to TiO(OH)H_2_PO_4_·2H_2_O and Ti(HPO_4_)_2_·H_2_O (not more than 10%) phases in the non-calcined sample according to thermolysis Equations (1) and (2) [33].

Thermolysis scheme

(1) $$\mathrm{TiO}(\mathrm{OH})(H_{2}{\mathrm{PO}}_{4})\cdot H_{2}O\frac{-H_{2}O}{20-180\;{}^{\circ}C}\to \mathrm{TiO}(\mathrm{OH})(H_{2}{\mathrm{PO}}_{4})\frac{-1.5H_{2}O}{180-760\;{}^{\circ}C}\to 0.5{\mathrm{Ti}}_{2}O({\mathrm{PO}}_{4}{)}_{2}\frac{\;}{650-880\;{}^{\circ}C}\to \;0.5{\left(\mathrm{TiO}\right)}_{2}P_{2}O_{7}$$

(2) $$\mathrm{Ti}({\mathrm{HPO}}_{4}{)}_{2}\cdot H_{2}O\;\frac{-H_{2}O}{70-250\;{}^{\circ}C}\;\to \;\mathrm{Ti}({\mathrm{HPO}}_{4}{)}_{2}\;\frac{-H_{2}O}{250-750\;{}^{\circ}C}\;\to \;{\mathrm{TiP}}_{2}O_{7}$$

(3) $$2\mathrm{TiO}(\mathrm{OH})({\mathrm{Ca}}_{0.5}{\mathrm{HPO}}_{4})\cdot H_{2}O\frac{-2H_{2}O}{20-130\;{}^{\circ}C}2\mathrm{TiO}(\mathrm{OH}){\mathrm{Ca}}_{0.5}{\mathrm{HPO}}_{4})\frac{-2H_{2}O}{130-180\;{}^{\circ}C}2\mathrm{TiO}({\mathrm{Ca}}_{0.5}{\mathrm{PO}}_{4})\frac{-H_{2}O}{280-640\;{}^{\circ}C}{\mathrm{CaTi}}_{2}O({\mathrm{PO}}_{4}{)}_{2}$$

(4) $$>820\;{}^{\circ}C:{2\mathrm{Ti}({\mathrm{HPO}}_{4})}_{2}+{\mathrm{CaTi}}_{2}O{\left({\mathrm{PO}}_{4}\right)}_{2}\to {\mathrm{CaTi}}_{4}{\left({\mathrm{PO}}_{4}\right)}_{6}$$

**Figure 2:**
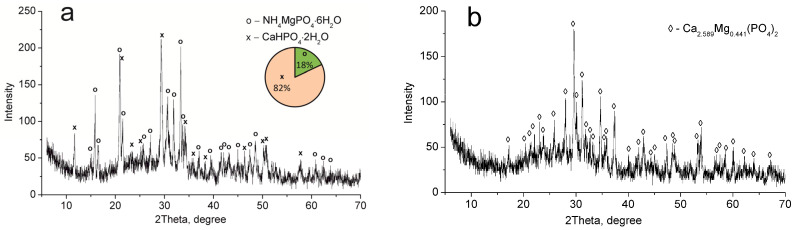
XRD patterns of the individual phases of calcium–magnesium phosphates synthesized (CMP sample): (**a**) 60 °C, (**b**) 850 °C.

The CMP sample dried at 60 °C consists of MgNH_4_PO_4_·6H_2_O and CaHPO_4_·2H_2_O phases (Figure 2a). Calcium and magnesium phosphate Ca_2_._589_Mg_0_._441_(PO_4_)_2_ have been established to be thermolysis products of these compounds after calcination at 850 °C (Figure 2b).

**Figure 3:**
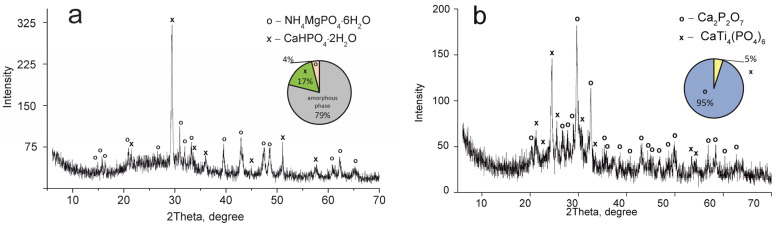
XRD patterns of the composite titanium, calcium and magnesium phosphates synthesized: (**a**) TCMP1 60 °C, (**b**) TCMP1 850 °C.

**Figure 4:**
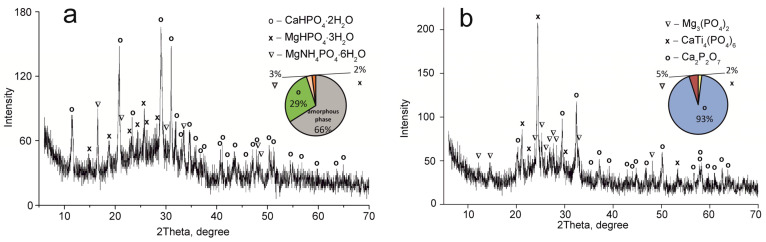
XRD patterns of the composite titanium, calcium and magnesium phosphates synthesized: (**a**) TCMP2 60 °C, (**b**) TCMP2 850 °C.

**Figure 5:**
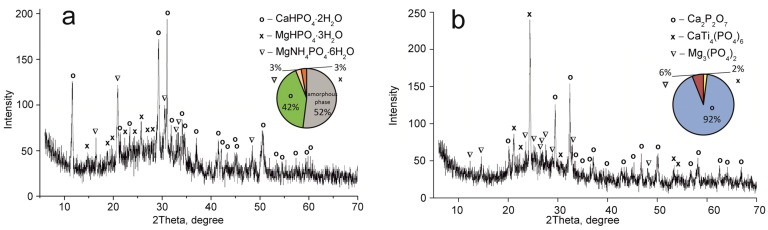
XRD patterns of the composite titanium, calcium and magnesium phosphates synthesized: (**a**) TCMP4 60 °C, (**b**) TCMP4 850 °C.

The elemental and phase composition of the materials obtained is shown in Table 1 and Figure 3, Figure 4 and Figure 5.

The CaTi_4_(PO_4_)_6_ phase was established to occur in all samples obtained. This fact indicates the presence of two phases of titanium phosphate TiO(OH)(H_2_PO_4_)·2H_2_O and Ti(HPO_4_)_2_·H_2_O in the composite sorbent (Equations (3) and (4)). The amount of titanium α-phosphate (Ti(HPO_4_)_2_·H_2_O) is obviously low since it has a crystal structure, though it was not found in the samples dried at 60 °C. The phase composition of calcium and magnesium phosphates is the same in all composite materials and represented by the following phases: CaHPO_4_·2H_2_O, MgHPO_4_·3H_2_O, and MgNH_4_PO_4_·3H_2_O. Diagrams in Figure 2, Figure 3, Figure 4 and Figure 5 illustrate phase proportion as detected by XRD analysis.

From the study of the textural properties of the materials obtained, the increase in the specific surface area, pore volume, and average pore diameter of samples was shown to increase with an increase in titanium phosphate share (Table 2). This occurs due to the amorphous nature of titanium phosphate. Adsorption–desorption isotherms obtained correspond to the IV type by IUPAC classification and indicate that all the samples are mesoporous materials.

**Figure 6:**
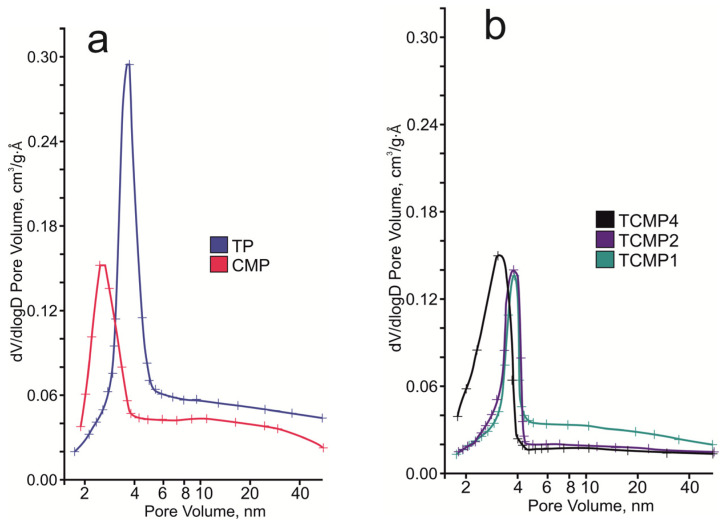
BJH desorption pore size distribution of the final solids: (**a**) TP, CMP; (**b**) TCMP1-4.

The pore system of the compounds synthesized consists of micro-, meso-, and macropores, according to the pore distribution curve (Figure 6). For samples containing calcium and magnesium phosphates as the predominant phases, a narrow peak is observed in the region of 3–3.5 nm. With an increase in the share of titanium phosphate in the sample, this peak shifts to the region of 4 nm.

**Figure 7:**
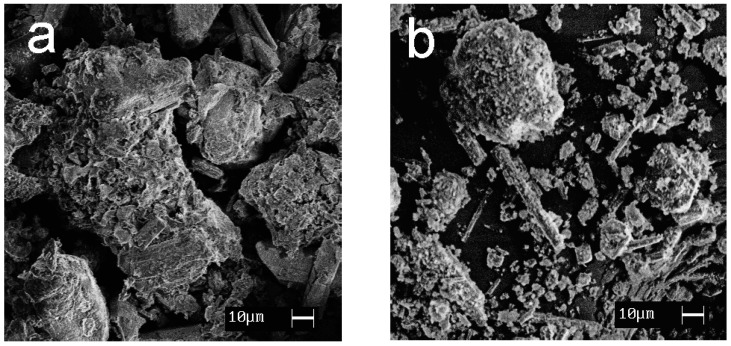

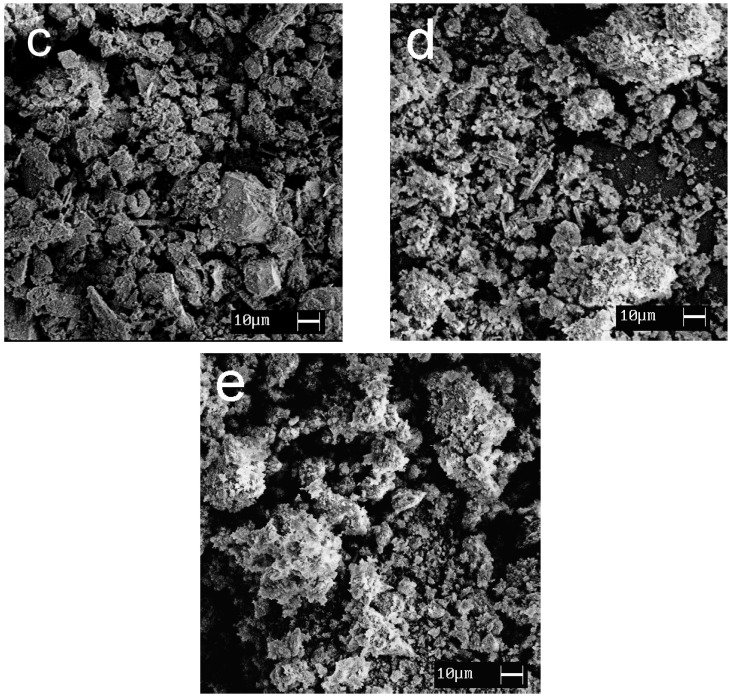
SEM images of the samples obtained: (**a**) CMP, (**b**) TCMP4, (**c**) TCMP2, (**d**) TCMP1, (**e**) TP.

SEM images showing the morphology of the synthesized materials are given in Figure 7. Calcium and magnesium phosphates are exhibited to be large crystalline particles with a size of 30–100 μm; titanium phosphates are characterized by the formation of agglomerates (10–30 μm) consisting of small, flat particles oriented in the same direction.

### 2.2. Sorption Study

The batch experiment was carried out to estimate sorption capacities. Sorbent (0.2 g) was added to an aqueous solution (40 mL). The pH was adjusted to the values of 3.0 ± 0.2 by adding 0.1 M HNO_3_ solution. The mixture was under stirring for 24 h. Then, the sorbent was removed using filtration. Sorption capacity was calculated according to Equation (5):

(5) $$q_{e}=\frac{\left(C_{i}-C_{e}\right)V}{m}$$

where *C_i_*—initial concentration of the element in the solution, mg·L^−1^; *C_e_*—equilibrium concentration of element after sorption, mg·L^−1^; *V*—solution volume, L; *m*—sorbent mass, g.

Data obtained were interpreted using the isotherm Equations (6)–(11) of the Langmuir, Freundlich, and Redlich–Peterson models.

Model

(6) $$\mathrm{Langmuir}\;\;\;\;\;\;\;q_{e}=\frac{q_{max}K_{L}C_{e}}{\left(1+K_{L}C_{e}\right)}$$

(7) Freundlich         qe=KFCe1n

(8) $$\mathrm{Redlich}–\mathrm{Peterson}\;\;\;\;\;\;\;q_{e}=\frac{K_{R}C_{e}}{\left(1+\alpha_{R}C_{e}^{\beta }\right)}$$

Linear form

(9) $$\mathrm{Langmuir}\;\;\;\;\;\frac{1}{q_{e}}=\frac{1}{q_{max}K_{L}C_{e}}+\frac{1}{q_{max}}$$

(10) $$\mathrm{Freundlich}\;\;\;\;\;\;\ln q_{e}=\ln K_{F}+\frac{1}{n}\ln C_{e}$$

(11) $$\mathrm{Redlich}–\mathrm{Peterson}\;\;\ln\left(K_{R}\frac{C_{e}}{q_{e}}-1\right)=\ln\alpha_{R}+\beta \ln C_{e}$$

where *C_e_*—equilibrium concentration of element after sorption, mg·L^−1^; *q_e_*—equilibrium sorption capacity, mg·g^−1^; *q_max_*—the value of the maximum sorption, mg·g^−1^; *K_L_*—the constant of the adsorption equilibrium (Langmuir constant) for monolayer sorption, characterizing the energy of the adsorbent–adsorbate bond, L·mg^−1^; *K_F_*—the constant of the adsorption equilibrium (Freundlich constant), characterizing the energy of the adsorbent–adsorbate bond (mg·g^−1^)(L·mg^−1^)^1/n^; *n*—an indicator of the heterogeneity of exchange centers, which characterizes the change in adsorption depending on the degree of centers filling; *K_R_* and *a_R_*—Redlich–Peterson constants, mL·g^−1^ and mL^β^·mg^−β^ (*K_R_*—the constant of the adsorption equilibrium for multilayer sorption); *β*—parameter characterizing the degree of heterogeneity of the sorbent surface indirectly, the value of *β* is in the range 0 < *β* < 1.

The Langmuir model is applicable in describing the process of sorption on a sorbent monolayer, wherein all sorption centers are identical and energetically equivalent. This model is suitable for comparing the sorption characteristics of different sorbents in case the model parameters indicate the adsorption intensity and the area of the sorbent’s open surface. The *K_L_* parameter correlates with the degree of porosity and the specific surface area of the sorbent.

The Freundlich isotherm is applicable to adsorption processes occurring on heterogenic surfaces. Parameters of this model characterize the heterogeneity of the surface and the exponential distribution of active centers and their energies. Additionally, the value of *1*/*n* expresses the relative distribution of energy and the heterogeneity of the sorption centers of the adsorbate.

The Redlich–Peterson equation includes three adjustable parameters in the isotherm equation and is widely used as a compromise between modeling by Langmuir and Freundlich. The equation matches the Langmuir isotherm if the value of *β* is equal to 1. When the value of the parameter *a_R_·Ceβ* is much greater than 1, the equation fits the Freundlich isotherm. The ratio *K_R_*/*a_R_* determines the degree of adsorption.

**Figure 8:**
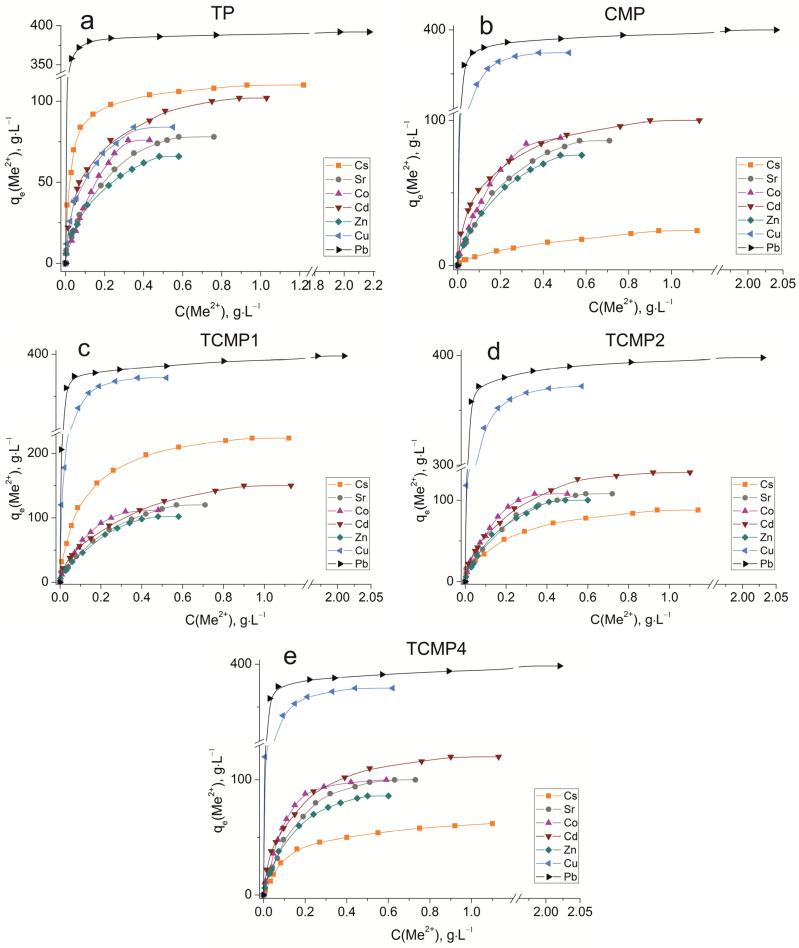
Sorption isotherms of Co^2+^, Cu^2+^, Cd^2+^, Cs^+^, Sr^2+^, Zn^2+^, and Pb^2+^ on the samples obtained: (**a**) TP, (**b**) CMP, (**c**) TCMP1, (**d**) TCMP2, (**e**) TCMP4.

**Figure A1:**
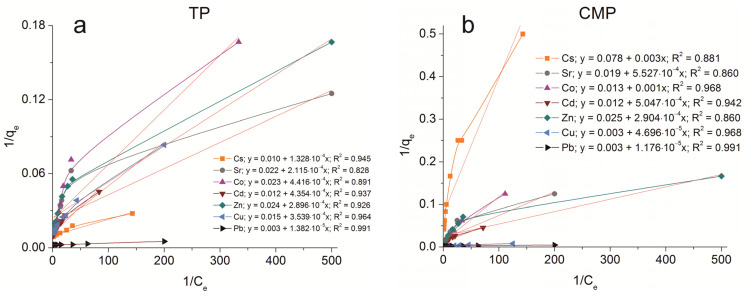

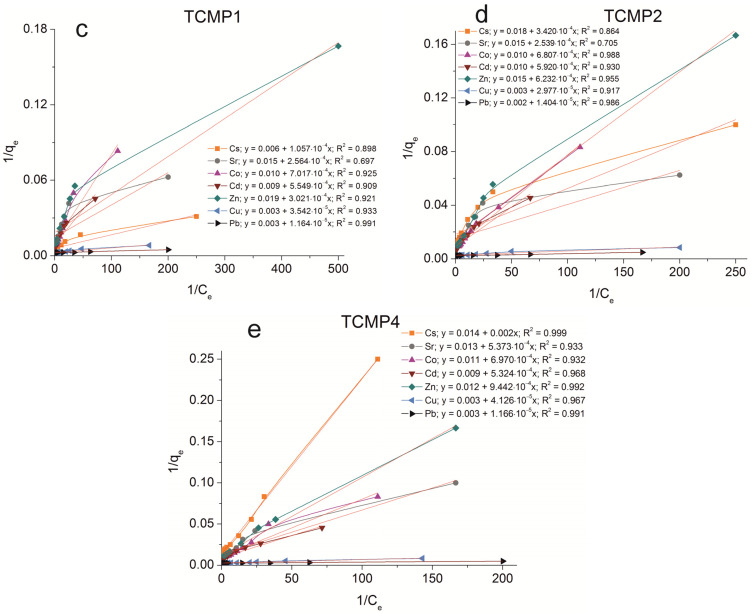
Langmuir isotherm plots of Cs^+^, Sr^2+^, Co^2+^, Cd^2+^, Zn^2+^, Cu^2+^, and Pb^2+^ sorption on the samples obtained: (**a**) TP, (**b**) CMP, (**c**) TCMP1, (**d**) TCMP2, (**e**) TCMP4.

**Figure A2:**
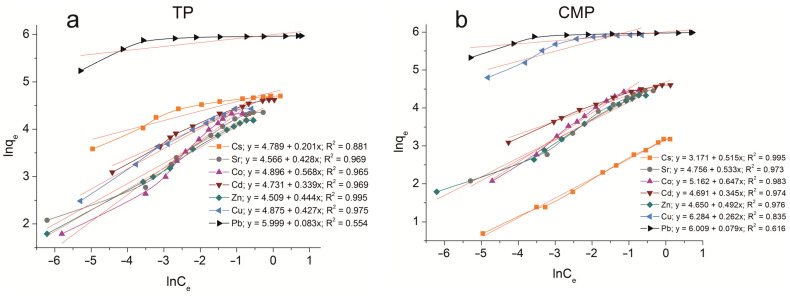

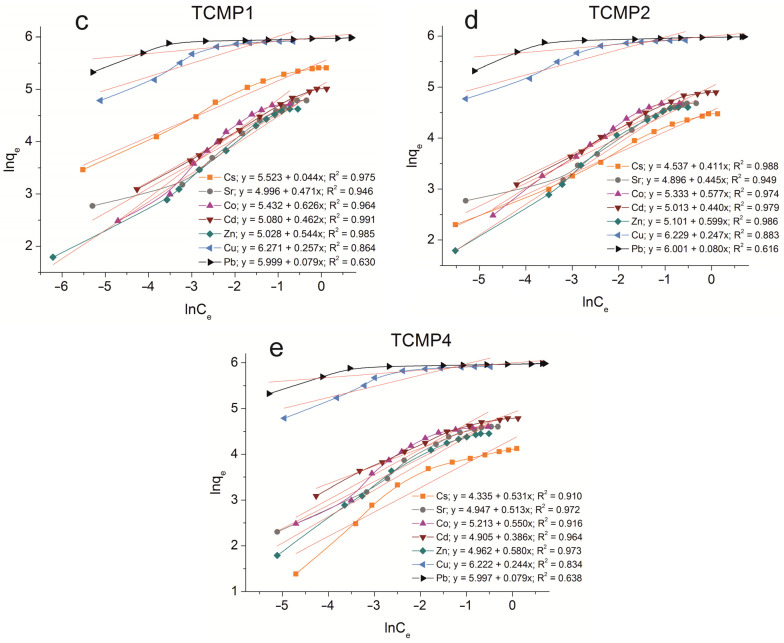
Freundlich isotherm plots of Cs^+^, Sr^2+^, Co^2+^, Cd^2+^, Zn^2+^, Cu^2+^, and Pb^2+^ sorption on the samples obtained: (**a**) TP, (**b**) CMP, (**c**) TCMP1, (**d**) TCMP2, (**e**) TCMP4.

**Figure A3:**
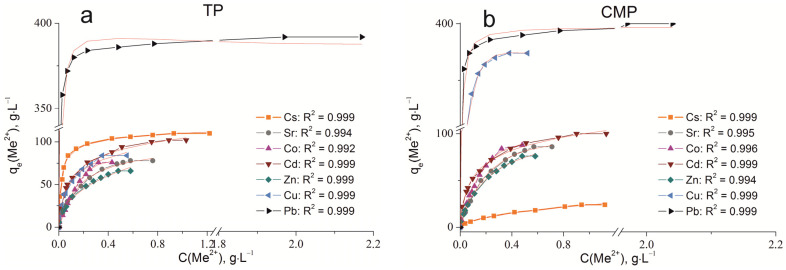

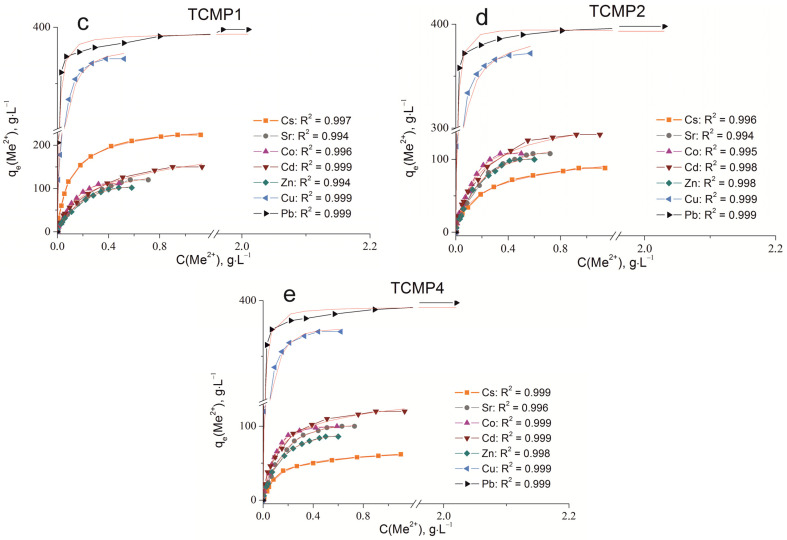
Redlich–Peterson fitting of isotherm plots of Cs^+^, Sr^2+^, Co^2+^, Cd^2+^, Zn^2+^, Cu^2+^, and Pb^2+^ sorption on the samples obtained: (**a**) TP, (**b**) CMP, (**c**) TCMP1, (**d**) TCMP2, (**e**) TCMP4.

The sorption isotherms were plotted for all samples obtained using the experimental sorption data for Cs^+^, Sr^2+^, Co^2+^, Cd^2+^, Zn^2+^, Cu^2+^, and Pb^2+^ cations (Figure 8). Parameters of equilibrium allow us to gain insight into the mechanism of the sorption process. To choose the most suitable model for describing the equilibrium of the sorption processes studied, the corresponding plots were plotted in the coordinates 1/*q_e_* vs. 1/*C_e_* and ln*q_e_* vs. ln*C_e_* for the Langmuir and Freundlich model, respectively (Figure A1 and Figure A2). Fitting of experimental data with the Redlich–Peterson model (Figure A3) was carried out using the Solver add-in in Microsoft’s spreadsheet, Microsoft Excel (version 2301) [37]. The parameters of all the models described are presented in Table A1, Table A2 and Table A3.

#### 2.2.1. Sorption on TP

TP (TiO(OH)H_2_PO_4_·H_2_O) refers to ion exchange materials in which protons of phosphate groups can be exchanged for metal cations. Experimental data show that complete removal of metals from solution is possible at initial concentrations of 0.9, 0.5, 0.25, 0.9, 0.4, 0.3, and 2.0, g·L^−1^ for Cs, Sr, Co, Cd, Zn, Cu, and Pb cations, respectively.

Phosphate functional groups are active if they are deprotonated. The dissociation constants of phosphate groups for the first and second dissociation steps are pK_1_ = 2.2 and pK_2_ = 7.1, respectively. The study of sorption of metal cations was carried out at pH = 3 to avoid the hydrolysis of copper, lead, and zinc, so ion exchange should proceed throughout the first step of dissociation under the chosen conditions. The theoretical ion exchange capacity of titanium phosphate, calculated by its compound formula (TiO(OH)H_2_PO_4_·H_2_O), is 9.35 meq·g^−1^, so in acid solutions, it can be 4.67 meq·g^−1^. The static exchange capacity for the cations studied is 110, 78, 76, 102, 65, 84, and 392 mg·g^−1^ for Cs, Sr, Co, Cd, Zn, Cu, and Pb, respectively.

The plots of isotherms obtained are of the H-type (high) for low hydrated cesium and lead cations and are characterized by a steep initial section of the isotherm in the region of low adsorbate concentrations. The high affinity of the metal ions abovementioned for titanium phosphate is associated with the size of their effective radii. These metals are characterized by large ionic radii and, thus, a small size of the hydration shell that facilitates the sorption without marked steric hindrances.

The sorption of all investigated ions is in good agreement with the Freundlich (R^2^ ≥ 0.99) and the Redlich–Peterson model (R^2^ > 0.99). This indicates that the sorption centers are not equivalent, and cations have difficulty occupying neighboring adsorption centers due to the repulsive forces acting when the surface is saturated.

#### 2.2.2. Sorption on CMP

The CMP is dissolved with the release of calcium, magnesium, and phosphate ions into a solution in the acidic pH region. Phosphates presented in the solution bind metal cations forming insoluble compounds. The dissolution of CMP is accompanied by an increase in the pH of the solution to values of 6–7.5, which is associated with decreasing H^+^ concentration due to the presence of producing H_2_PO_4_^–^-species, according to the following reaction:

2(Ca,Mg)HPO_4_ + 2H^+^ ↔ 2(Ca,Mg)^+^ + H_2_PO_4_^–^

The release of phosphate ions into solution leads to the formation of insoluble metal phosphates according to the reaction:

2H_2_PO_4_^–^ + 3Me^2+^ → Me_3_(PO_4_)_2_ + 4H^+^

Obviously, for all metals studied, their rapid precipitation in the form of insoluble phosphates is observed, except for cesium. Additionally, the process of precipitation as insoluble hydroxides is observed for zinc and copper due to their pH at the initial stage of metal hydroxide precipitation. The solubility of metal phosphates and the pH of the initial stage of metal hydroxide precipitation are given in Table 3.

The considerable excess of the phosphate ion in the solution proceeds due to the formation of insoluble copper phosphates (Cu_3_(PO_4_)_2_) at low initial concentrations of copper. With an increase in copper concentration in the initial solution, the precipitation of copper hydroxides participates in the immobilization process, as the final pH value of the solution is 7.43. At the same time, copper hydrolysis products in the form of Cu(OH)^+^ species can occur in the solution. Previously, we reported that pH_zpc_ (pH of zero point charge) for CMP is 6.13 [35]. This means that at higher pH values, the sorbent surface is charged negatively, so electrostatic interaction of hydrolyzed particles having a positive charge with the sorbent surface is possible. Thus, the removal of copper from acidic solutions proceeds according to three mechanisms: precipitation of copper phosphates, precipitation of copper hydroxides, and surface complexation. At the same time, the amount of copper removed from the solution (374 mg·g^−1^) is much greater than that removed by sorption on titanium phosphate according to the ion exchange mechanism (84 mg·g^−1^). Isotherms of copper sorption on CMP fit the Freundlich and Redlich–Peterson isotherms (R^2^ = 0.999). An assumption about the equipotential distribution of sorption centers is made for the Freundlich model. Obviously, large hydrolyzed copper particles cannot occupy neighboring deprotonated centers due to the action of mutual repulsion forces. Moreover, accordance of the experimental data with the Freundlich model confirms that the contribution of complexation to the overall mechanism of sorption is quite low.

The same complex metal removal mechanism can be expected for lead and zinc cations since the final pH values in the system are 7.44 and 6.8, respectively. The amount of phosphate groups released into the solution from CMP is sufficient for the complete binding of lead and zinc into insoluble phosphates. The amounts of adsorbed lead and zinc are 400 mg·g^−1^ and 107 mg·g^−1^, respectively. According to the data obtained, only the Pb_3_(PO_4_)_2_ phase is recorded in the XRD spectra after lead sorption [35]. The constant of the solubility product of this compound is 10^–43^, which confirms the conversion of lead into insoluble phosphate. The constant of the solubility product of zinc phosphate is markedly lower. Therefore, at pH > 5.5, Zn(OH)^+^ is present in the solution, along with Zn^2+^. The zinc hydroxide precipitation starts at a pH of 6.4, so the small part of zinc precipitates as hydroxide, and hydrolyzed species interact with sorption centers charged negatively, according to the mechanism of surface complexation. The amount of copper sorbed on the CMP sample was observed to be twice greater than that of zinc. This occurs due to the higher share of copper precipitation as hydroxide. For zinc cations, a large amount of hydrolyzed forms is quite probable. These particles are difficult to remove by the mechanism of surface complexation at high initial metal concentrations. The sorption process of Zn^2+^ cations is well described by the Redlich–Peterson and Freundlich models (R^2^ > 0.99), as that for Pb^2+^ (R^2^ = 0.999).

For less hydrolyzed cations, such as Cd^2+^, Co^2+^, and Sr^2+^, the final pH value in solution (7.16–7.4) should not have a marked effect on the state of these metals in solution. Therefore, metal cations are presented in the solution in the form of unhydrolyzed species. Experiments have demonstrated that 86 mg·g^−1^ Co, 100 mg·g^−1^ Cd, and 84 mg·g^−1^ Sr are removed from the solutions. K_sp_ values of Sr, Cd, and Co are 10^−31^, 10^−33^, and 10^−35^, respectively. This allows the precipitation of metal phosphates in the solutions studied. The ionic radii of Cd, Sr, and Co are 99, 112, and 78 pm, respectively. The ionic radii of Ca and Mg are 99 and 78 pm, respectively. Therefore, Cd can replace Ca in the crystal structure, and Co can substitute for both calcium and magnesium. In the sorption of Sr on the CMP sample, ion exchange reactions are not possible due to the large ionic radius. The sorption equilibrium of strontium is adequately described by the Redlich–Peterson model (R^2^ > 0.99). The Freundlich model (R^2^ = 0.999) is the most relevant model for describing the sorption process of cadmium and cobalt.

The solubility of cesium phosphate is comparable to that of calcium and magnesium phosphates. Thus, the formation of insoluble Cs_3_PO_4_ is unlikely. Cesium also cannot substitute the structure-forming CMP cations due to its large ionic radius (186 pm) and form insoluble hydroxides under the chosen conditions (the equilibrium pH value in the system is 7.4). Therefore, CMP cannot be an effective sorbent for removing Cs^+^ from aqueous media, and, indeed, the maximum value of its sorption capacity is 22 mg·g^−1^. The sorption equilibrium of cesium is most adequately described by the Redlich–Peterson model (R^2^ = 0.999).

Hydroxide precipitation has been established to be the main mechanism of copper removal. Lead and zinc can be removed from solutions due to insoluble phosphate precipitation as the main removal process. Sorption of cesium on CMP proceeds according to the ion exchange only. The complex mechanism of metal removal is observed for Cd, Co, and Sr. The process of cobalt and cadmium removal consists of phosphate precipitation, surface complexation, and ion exchange. Strontium can be removed due to phosphate precipitation and surface complexation. The sorption of cations studied on CMP proceeds according to a complex mechanism, the selectivity of the sorbent towards ions studied can be estimated only by the metal amount removed from the solution (mg·L^−1^).

There is reason to assume that sorption efficiency on composite materials will be higher due to the synergism of individual phosphates. To confirm this assumption of sorption equilibria on TCMP, different ratios of titanium phosphate to calcium–magnesium phosphates have been studied.

#### 2.2.3. Sorption on TCMPs

The ion exchange mechanism contributes to the sorption process for lead cations since the amount of lead removed remains actually unchanged with a decrease in the CMP share in the compound. The composition of the sorbent has no effect on the level of removal of readily hydrolyzable copper cations. Even for TCMP1, where the share of titanium phosphate is 50%, the final pH value of the solutions is 6.8. From the calculations, it is obvious that only 16% of total copper (374 mg·g^−1^) is removed as copper phosphates and 84% as hydroxide. Sorption isotherms of Pb and Cu on composite sorbents are described in the same way as on CMP by the Freundlich and Redlich–Peterson equations (R^2^ = 0.999).

The absorption of zinc decreases with an increase in the titanium phosphate share in the TCMP samples. The pH value of the solution at equilibrium is 5.28 for TCMP1, and it is lower than that of the beginning of metal hydroxide precipitation. Therefore, at low values of the initial concentration of zinc in solution, the main mechanism of zinc removal is phosphate precipitation because precipitation is a faster process than ion exchange. An increase in the concentration of zinc in solution leads to an increasing role of the ion-exchange mechanism in the overall sorption process. The synergism of the individual phases is expressed in the increased sorption capacity of the composite material (102 mg·g^−1^ for TCMP1) compared to the TP. It should be concluded that the sorption capacity of the composite materials increases with an increase in the titanium phosphate share due to the increased contribution of the ion exchange mechanism in metal sorption. The addition of CMP into the material promotes an increase in the final pH of the solution, which in turn promotes ion exchange sorption on titanium phosphate. With the increase in CMP share in the final product, zinc phosphate precipitation and surface complexation processes intensify metal removal. The final pH of the system is 6.32 and 6.8 for TCMP2 and TCMP4, respectively. The presence of hydrolyzed forms of zinc cations in such solutions hinders their sorption by the ion exchange mechanism. Therefore, the amounts of metal removed from the solution decrease and make up 98 mg·g^−1^ and 84 mg·g^−1^ for TCMP2 and TCMP4, respectively. The results obtained are adequately interpreted by the Freundlich and Redlich–Peterson models (R^2^ > 0.99).

In the sorption of cobalt, cadmium, and strontium on a composite sorbent, the final pH values for all solutions are lower than the pH of the initial stages of metal hydroxide precipitation (Table 3). Therefore, the mechanism of cation removal from the solution is controlled by the phase ratio in the composite sorbent. With an increase in the share of TP in the sample, the amount of metal removed gradually increases from 98–120 mg·g^−1^ on TCMP4 to 112–150 mg·g^−1^ on TCMP1. Cadmium removal proceeds according to ion exchange on calcium–magnesium phosphates and cadmium-insoluble phosphate precipitation. For strontium, the main mechanism is phosphate precipitation. The most proper model for Cd and Sr sorption equilibrium describing is the Redlich–Peterson model (R^2^ > 0.99). The ion exchange mechanism of both titanium phosphate and calcium–magnesium phosphate contributes markedly to the cobalt sorption mechanism; the equilibrium isotherms are adequately described by the Freundlich (R^2^ > 0.99) and Redlich–Peterson equations (R^2^ = 0.999).

**Figure 9:**
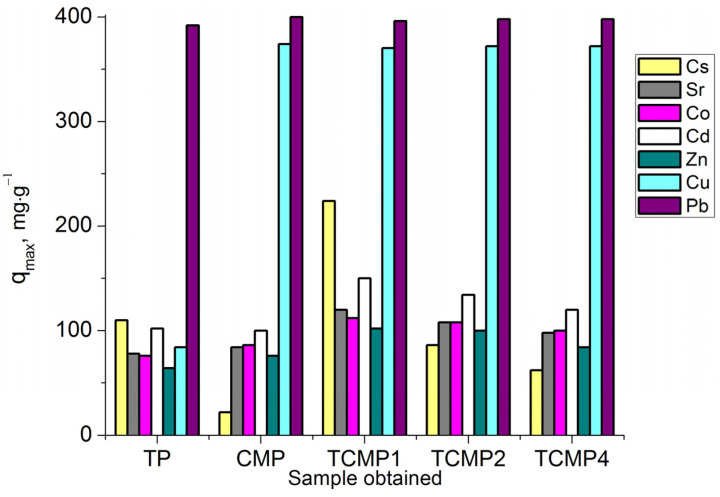
Sorption capacity on TP, CMP, TCMP1-4 towards Cs^+^, Sr^2+^, Co^2+^, Cd^2+^, Zn^2+^, Cu^2+^, and Pb^2+^.

The sorption process of Cs^+^ cations on all sorbent samples can be most properly described using the Redlich–Peterson model (R^2^ > 0.99). The amount of metal removed from the solution increases with an increase in titanium phosphate’s share in the composite sorbent. The values of sorption capacities *q_max_* (mg·g^−1^) for the samples synthesized: TP, TCMP1-4, and CMP are presented in Figure 9.

In order to check the accuracy of the experimental data, the coefficient of determination (*R*-squared) *R*^2^ (12) was calculated by using the following equation:

(12) $$R^{2}=\frac{\sum {\left(q_{e,exp}-{\bar{q}}_{e,exp}\right)}^{2}-\sum {\left(q_{e,exp}-q_{e,calc}\right)}^{2}}{\sum {\left(q_{e,exp}-{\bar{q}}_{e,exp}\right)}^{2}}$$

where *q_e,calc_*—the equilibrium capacity calculated from the isotherm equation, mg·g^−1^; *q_e,exp_* and *q_ē,exp_*—the equilibrium capacity received experimentally and average value of *q_e,exp_*, mg·g^−1^ [37]. The goodness of fitting experimental data with models considered was confirmed by the highest *R*-squared values (Table A1, Table A2 and Table A3).

All the composite sorbents studied compared with individual phosphates are found to demonstrate a marked increase in the level of their efficiency in the purification of solutions due to the synergism of the components of new materials. Composite sorbent with molar ratio Ti:(Ca+Mg) of 1:1 (TCMP1) is the optimal material for removing the tested ions from solutions.

#### 2.2.4. Sorption Test

Sorption of ^137^Cs, ^90^Sr and ^60^Co radionuclides from multicomponent sewage water with pH of 6.2 and salinity of 4.5 g·L^−1^ was studied in a batch mode at 20 °C, the ratio of solid and liquid phase *V*/*m* was 250 mL·g^−1^ with a contact time of 24 h. The activity of radionuclides in sewage water (*A_init_*) was as follows: 10^5^ Bq·L^−1^: ^137^Cs 45.025, ^90^Sr 40.178, ^60^Co 8.350. Removal efficiency (*S*, %) and distribution coefficient (*K_d_*, mL·g^−1^) were calculated by the following equations:

(13) $$S=\frac{A_{init}-A_{eq}}{A_{init}}\cdot 100\%$$

(14) $$K_{d}=\frac{A_{init}-A_{eq}}{A_{eq}}\cdot \frac{V}{m}$$

where *A_init_*—activity of radionuclides before sorption, Bq·L^−1^; *A_eq_*—activity of radionuclides after sorption, Bq·L^−1^; *V* is the volume of solution, mL; *m*—the mass of the sorbent, g.

The activity of radionuclides after purification and sorption characteristics of sorbents studied are given in Table 4.

Batch experiments demonstrated the high efficiency of composite sorbents TCMP1-4 in multicomponent LRW purification. The behavior of ^137^Cs, ^90^Sr, and ^60^Co radionuclides in sorption on sorbents synthesized is similar to that of their non-radioactive analogs. The distribution coefficient for all the radionuclides studied is found to be more than 10^5^ mL·g^−1^.

## 3. Materials and Methods

### 3.1. Starting Materials

Titanium salt ((NH_4_)_2_TiO(SO_4_)_2_·H_2_O)-ATS was used as a titanium precursor. This salt can be produced as a by-product from titanium-containing ore. ATS was obtained from the mineral titanite (CaTiSiO_5_), which is an unprocessed industrial waste of apatite–nepheline ores processing [34]. The chemical composition of the salt was found to be as follows, % wt.: TiO_2_—19.5, NH_4_^+^—9.7, SO_4_^2–^—52.3. Calcined (850 °C) dolomite (CaCO_3_·MgO) was used as the precursor of calcium and magnesium [33]. The elemental composition of CD was as follows, % wt.: Ca—18.59; Mg—11.27; Si—3.78; K—1.08; S—0.74; Ti—0.24; Fe—2.44; Mn—0.12; Cl—0.31.

Metal salts (Pb(NO_3_)_2_, Zn(NO_3_)_2_·6H_2_O, Co(NO_3_)_2_·6H_2_O, Sr(NO_3_)_2_, Cu(NO_3_)_2_·3H_2_O, Cd(NO_3_)_2_·4H_2_O, and CsNO_3_) for preparing the solutions, and H_3_PO_4_, HF, HCl, and HNO_3_ were purchased from Neva-Reaktiv (Saint-Petersburg, Russia). Deionized water for dissolving the salts and the blue-band filter paper for filtration of the suspensions were used in all experiments. All the chemicals were of analytical reagent grade and were used without preceding purification.

### 3.2. TP, TCMP1-4, and CMP Synthesis

Synthesis of composite sorbents (TCMP1-4) was carried out according to a method previously developed by the authors [35]. Synthesis is based on green chemistry principles, when one product formed previously during the synthesis is a precursor for obtaining the final product. This allows for simplifying the chemical interaction of the components following their stoichiometric consumption considerably. A 10% phosphoric acid was used as a precipitant. Molar ratio Ti:(Ca+Mg) was 1:1 ÷ 4. To compare the sorption characteristics of composite materials obtained with individual phosphates, TP (titanium phosphate) and CMP (calcium–magnesium phosphates) were synthesized.

For the TP synthesis, 10 g of (NH_4_)_2_TiO(SO_4_)_2_·H_2_O was mixed with 45.4 mL 1.12 M H_3_PO_4_. The obtained suspension was heated to 60 °C for 5 h under stirring.

For the TCMP1-4 synthesis, 10 g of (NH_4_)_2_TiO(SO_4_)_2_·H_2_O was mixed with 45.4 mL 1.12 M H_3_PO_4_. The amount of phosphorus in the mixture was enough to achieve the molar ratio of Ti:P = 1:4. The obtained suspension was heated to 60 °C for 5 h under stirring. The reaction between the titanium salt and phosphoric acid was accompanied by titanium salt dissolving, followed by titanium phosphate precipitation. The excess of phosphoric acid in the reaction system provided the formation of a buffer (NH_4_H_2_PO_4_+H_3_PO_4_) with pH = 2. Then for TCMP1, TCMP2, and TCMP4, 10.0, 18.7, and 37.4 g of calcined dolomite was loaded into the suspension, respectively. These quantities correspond to the molar ratios (Ca+Mg):P of 1:1, 1:2 and 1:4 for TCMP1, TCMP2, and TCMP4, respectively. The mixtures were stirred at 60 °C for 3 h. The buffer prevented the formation of a soluble form of calcium and magnesium phosphates and ensured the complete conversion of calcium and magnesium into phosphates.

For the synthesis of CMP, 17.6 mL 1M NH_4_H_2_PO_4_ solution was heated to 60 °C, and 10 g of calcined dolomite was gradually added. The molar ratio (Ca+Mg):P was 1:1. The suspension was kept under constant stirring for 3 h.

All resulting solids were separated from the liquid phase by filtration, washed with H_2_O to a pH of ~6, and dried at 60 °C. Synthesis conditions are shown in Table 5.

### 3.3. Characterization Techniques

For elemental analysis, solid samples were dissolved in a mixture of acids (HF, HNO_3_, and HCl), then Ti, Ca, Mg, and P were determined using an ICPE-9000 inductively coupled plasma atomic emission spectrometer (Shimadzu Corporation, Tokyo, Japan). Sulfur was determined gravimetrically using barium sulfate. Cs, Sr, Co, Cd, Zn, Cu, and Pb solutions before and after sorption were analyzed using the atomic adsorption method on a PerkinElmer AAS 300 spectrometer (PerkinElmer Inc., Shelton, CT, USA). All elemental analysis data were obtained with an accuracy of ±0.05%.

A Shimadzu D6000 diffractometer (Shimadzu Corporation, Japan) with a monochrome CuKα study (λ = 1.5418 Å) was employed for obtaining XRD data. The pH measurements were performed using LAB 850 glass electrode pH meter (SI Analytics, Mainz, Germany). Radionuclides activity in the solutions before and after sorption was measured with a γ-spectrometer Canberra (Canberra Industries, Meriden, CT, USA) with a germanium detector. Strontium activity was measured with a Tesla low-background flow-proportional counter. A Tristar 320 surface analyzer (Micromeritics Company, Norcross, GA, USA) and low-temperature nitrogen adsorption method were used for the textural characteristic of final solids. The solid sample was degassed at 60 °C for 24 h before measurement. Pore size was calculated using the Barrett-Joyner-Halenda (BJH) method. SEM images were obtained using a scanning electron microscope SEMLEO-420 (Carl Zeiss, Oberkochen, Germany).

## 4. Conclusions

The composite sorbents based on titanium, calcium, and magnesium phosphates with various phase ratios Ti:(Ca+Mg) have been synthesized. The sorption properties on samples obtained toward Cs^+^, Sr^2+^, Co^2+^, Cd^2+^, Zn^2+^, Cu^2+^, and Pb^2+^ have been studied. The sorption of cations studied from initial solutions with a pH value of 3 has been studied. The adsorption isotherms on the sorbents obtained towards metal ions studied were analyzed using the Langmuir, Freundlich, and Redlich–Peterson models.

The sorption of all ions studied on TP fits the Freundlich and Redlich–Peterson models. The sorption centers on the sorbent surface are not equivalent, and cations are difficult to occupy the neighboring adsorption center due to the action of repulsive forces as the surface is filled.

The dissolution/precipitation mechanism was found to be the dominant mechanism for Co^2+^, Cd^2+^, Sr^2+^, Zn^2+^, and Pb^2+^ on CMP sorbent. CMP dissolution with release of phosphate into solution facilitates the precipitation of metal phosphates due to the difference in solubility between the Ca-Mg phosphates and phosphates of the metals to be removed. The high sorption ability of Cu ions, which is the most hydrolyzable cation among studied ones, is mainly determined by copper hydroxide precipitation. Lead sorption is likely to proceed by precipitation of insoluble phosphate. Zinc sorption proceeds due to both the dissolution/precipitation and surface complexation mechanisms. Isotherms of copper, lead, and zinc sorption on CMP fit the Freundlich and Redlich–Peterson isotherms.

The sorption of strontium proceeds due to the precipitation of insoluble phosphates. The most proper model, in this case, is the Redlich–Peterson one. For cadmium and cobalt, as the least hydrolyzable ions, the sorption mechanism is complex and composed of insoluble phosphate precipitation, ion exchange, and surface complexation mechanism. For these ions, the sorption equilibrium is adequately described by the Freundlich and Redlich–Peterson models.

The composition of the composite sorbent has no effect on the level of sorption of readily hydrolyzable lead and copper cations. Sorption isotherms can be described according to the Freundlich and Redlich–Peterson models.

The sorption of cesium proceeds according to ion exchange only. The sorption capacity of cesium cations on titanium phosphate in acid solutions is determined by the value of the theoretical ion exchange capacity of titanium phosphate. It is 4.67 meq·g^−1^ in acid solutions, and maximal (9.35 meq·g^−1^) in solutions with a pH range of >7. The higher values of pH of solutions (6–7.5) after the addition of composite sorbent are caused by the presence of Ca-Mg phosphates. Therefore, the sorption efficiency increases in the composite sorbents. The most proper model for the equilibrium of Cs cations is the Redlich–Peterson one.

For Cd, Co, Sr, and Zn cations, the sorption efficiency on composite materials synthesized is established to increase with the increase in titanium phosphate’s share in the sample. It is caused by the increase in the contribution of ion exchange processes. The sorption of zinc proceeds according to phosphate precipitation, ion exchange, and surface complexation. Zinc isotherms are described by the Freundlich and Redlich–Peterson models. Cadmium sorption proceeds according to ion exchange and insoluble cadmium phosphate precipitation. For strontium, the main mechanism is phosphate precipitation. The most proper model for Cd and Sr sorption equilibrium is the Redlich–Peterson model. The equilibrium of the sorption process of Co cations fits the Freundlich and Redlich–Peterson models.

The presence of different types of functional groups in the composite sorbent ensures its higher sorption capacity toward the cations studied. An easier synthesis procedure allows the production of a sorbent with an adapted composition for the treatment of various wastewaters.

## Figures and Tables

**Table 1:** Composition of samples and the degree of conversion of Ca and Mg into phosphate compounds.

Sample	Component, mas. %						Degree of Conversion, %		
	Ti	P	NH_4_^+^	S	Ca	Mg	Ti	Ca	Mg
TP	21.90	14.82	1.98	0.02	–	–	99.9	–	–
TCMP1	11.00	15.33	0.12	0.14	7.12	1.76	99.8	98.3	96.2
TCMP2	7.42	15.45	0.21	0.35	9.73	2.38	99.8	98.4	96.3
TCMP4	4.39	15.80	0.22	0.28	11.60	2.78	99.8	98.5	96.3
CMP	–	16.15	–	0.34	14.81	3.77	–	98.9	97.1

**Table 2:** Surface properties of the samples synthesized.

Sample	S, m^2^·g^−1^	Pore Volume, cm^3^·g^−1^	Pore Diameter, nm
TP	124.48	0.41	11.23
TCMP1	66.88	0.24	14.15
TCMP2	51.35	0.13	12.58
TCMP4	45.78	0.10	8.52
CMP	34.27	0.09	5.68

**Table 3:** Solubility product constants *K_sp_* at 25 °C and pH of the initial stage of metal hydroxide precipitation.

Formula	*K_sp_*	Formula	pH of the Initial Stage of Hydroxide Precipitation
Cd_3_(PO_4_)_2_	2.5·10^−33^	Cd(OH)_2_	8.3
Co_3_(PO_4_)_2_	2.1·10^−35^	Co(OH)_2_	8.2
Cs_3_PO_4_	1.1·10^−7^	CsOH	13.0
Cu_3_(PO_4_)_2_	1.4·10^−37^	Cu(OH)_2_	5.2
Pb_3_(PO_4_)_2_	7.9·10^−43^	Pb(OH)_2_	6.4
Sr_3_(PO_4_)_2_	1.0·10^−31^	Sr(OH)_2_	8.5
Zn_3_(PO_4_)_2_	9.1·10^−33^	Zn(OH)_2_	6.4
CaHPO_4_	2.7·10^−7^		
MgHPO_4_	0.2·10^−5^		
MgNH_4_PO_4_	2.5·10^−13^		

**Table 4:** Sorption characteristics of sorbents synthesized.

Sample	Activity of Radionuclides *A_eq_*, 10^5^ Bq·L^−1^			Distribution Coefficient *K_d_*, 10^5^ mL·g^−1^			Removal Efficiency *S*, %		
	^137^Cs	^90^Sr	^60^Co	^137^Cs	^90^Sr	^60^Co	^137^Cs	^90^Sr	^60^Co
TP	3.20·10^−2^	4.67·10^−2^	2.76·10^−2^	3.52	2.15	0.754	99.93	99.88	99.67
TCMP1	1.80·10^−2^	3.94·10^−2^	2.52·10^−2^	6.25	2.55	0.826	99.96	99.90	99.70
TCMP2	4.35	3.52·10^−2^	9.31·10^−3^	2.34·10^−2^	2.85	2.24	90.34	99.91	99.89
TCMP4	20.46	1.28·10^−2^	1.11·10^−3^	3.00·10^−3^	7.84	18.80	54.55	99.97	99.99
CMP	41.71	5.16·10^−3^	5.45·10^−4^	2.65·10^−4^	19.46	38.30	9.58	99.99	99.99

**Table 5:** Synthesis conditions of TP, TCMP1-4, CMP.

Sample	Ti:P (Molar)	(Ca+Mg):P (Molar)	Ti:(Ca+Mg) (Molar)	Time, h
TP	1:1	–	–	5
TCMP4	1:1	1:1	1:4	5 + 3 *
TCMP2	1:1	1:1	1:2	5 + 3 *
TCMP1	1:1	1:1	1:1	5 + 3 *
CMP	–	1:1	–	3

* 5 h for the first stage—TP synthesis, 3 h for the second stage—CMP synthesis.

## Data Availability

Data sharing is not applicable.

## Appendix A

The coefficient of determination *R*^2^ shown in Table A1, Table A2 and Table A3 was calculated according to Equation (12).

**Table A1:** Langmuir sorption isotherm constants for the sorption of Cs^+^, Sr^2+^, Co^2+^, Cd^2+^, Zn^2+^, Cu^2+^, and Pb^2+^ on the samples obtained.

Cation	*K_L_*	*q_e,calc_* *, mg·g^−1^	*q_e,exp_* *, mg·g^−1^	*R* ^2^
TP				
Cs	75.30	100	110	0.984
Sr	104.02	46	78	0.976
Co	52.08	44	76	0.979
Cd	27.56	84	102	0.979
Zn	82.87	42	66	0.983
Cu	42.38	67	84	0.983
Pb	217.08	334	392	0.915
CMP				
Cs	26.00	13	24	0.975
Sr	34.38	53	86	0.973
Co	13.00	77	88	0.982
Cd	23.78	84	100	0.985
Zn	86.09	40	76	0.974
Cu	63.88	334	374	0.928
Pb	255.10	334	400	0.916
TCMP1				
Cs	56.76	167	224	0.989
Sr	58.50	67	120	0.974
Co	14.25	100	112	0.976
Cd	16.22	112	150	0.982
Zn	62.89	53	102	0.969
Cu	84.70	334	372	0.954
Pb	257.73	334	398	0.916
TCMP2				
Cs	52.63	56	88	0.979
Sr	59.08	67	108	0.978
Co	14.69	100	108	0.972
Cd	16.89	100	134	0.966
Zn	24.07	87	100	0.999
Cu	100.77	334	372	0.971
Pb	142.45	500	398	0.919
TCMP4				
Cs	7.00	71	62	0.991
Sr	24.20	77	100	0.975
Co	15.78	91	100	0.961
Cd	16.90	112	120	0.997
Zn	12.71	83	86	0.962
Cu	72.71	334	372	0.938
Pb	257.29	334	398	0.916

* *q_e,calc_*—the equilibrium capacity calculated from the isotherm equation; *q_e,exp_*—the equilibrium capacity received experimentally.

**Table A2:** Freundlich isotherms constants for the sorption of Cs^+^, Sr^2+^, Co^2+^, Cd^2+^, Zn^2+^, Cu^2+^, and Pb^2+^ on the samples obtained.

Cation	*K_F_*	*n*	*R* ^2^
TP			
Cs	120.18	4.98	0.999
Sr	96.16	2.34	0.998
Co	133.75	1.76	0.997
Cd	113.41	2.95	0.999
Zn	90.83	2.25	0.999
Cu	130.97	2.34	0.999
Pb	403.02	12.05	0.999
CMP			
Cs	23.83	1.94	0.999
Sr	116.28	1.88	0.937
Co	174.51	1.55	0.999
Cd	108.96	2.90	0.999
Zn	104.58	2.03	0.995
Cu	535.93	3.82	0.999
Pb	407.07	12.66	0.999
TCMP1			
Cs	250.38	22.73	0.936
Sr	147.82	2.12	0.996
Co	228.61	1.60	0.999
Cd	160.77	2.16	0.999
Zn	152.63	1.84	0.999
Cu	529.00	3.89	0.999
Pb	403.02	12.66	0.999
TCMP2			
Cs	93.41	2.43	0.999
Sr	113.75	2.25	0.943
Co	207.06	1.73	0.999
Cd	150.35	2.27	0.999
Zn	164.19	1.67	0.999
Cu	507.25	4.05	0.999
Pb	403.83	12.50	0.999
TCMP4			
Cs	76.32	1.88	0.999
Sr	140.75	1.95	0.999
Co	183.64	1.82	0.999
Cd	134.96	2.59	0.999
Zn	142.88	1.72	0.999
Cu	503.71	4.10	0.999
Pb	402.22	12.66	0.999

**Table A3:** Redlich–Peterson isotherms constants for the sorption of Cs^+^, Sr^2+^, Co^2+^, Cd^2+^, Zn^2+^, Cu^2+^, and Pb^2+^ on the samples obtained.

Cation	*K_R_*	*a_R_*	*β*	*R* ^2^
TP				
Cs	7050	62.95	0.944	0.999
Sr	584	5.92	1.003	0.994
Co	407	5.24	1.683	0.992
Cd	2163	19.74	0.856	0.999
Zn	4438	49.47	0.604	0.999
Cu	2162	20.70	0.838	0.999
Pb	71,106	181.26	1.011	0.999
CMP				
Cs	2667	110.66	0.498	0.999
Sr	440	3.84	1.177	0.995
Co	526	4.81	1.316	0.996
Cd	1984	18.64	0.842	0.999
Zn	583	5.49	0.919	0.994
Cu	15,912	42.17	1.058	0.999
Pb	85,160	214.04	1.001	0.999
TCMP1				
Cs	2868	11.75	0.950	0.997
Sr	650	3.90	1.040	0.994
Co	730	6.17	1.464	0.996
Cd	1260	7.34	0.804	0.999
Zn	606	4.43	1.158	0.994
Cu	18,301	47.33	1.022	0.999
Pb	86,965	220.29	1.000	0.999
TCMP2				
Cs	890	9.24	0.851	0.996
Sr	649	4.60	1.086	0.994
Co	787	6.46	1.328	0.995
Cd	1040	6.64	0.941	0.998
Zn	630	4.93	1.182	0.998
Cu	21,987	55.83	0.985	0.999
Pb	72,780	183.56	1.008	0.999
TCMP4				
Cs	548	8.07	0.989	0.999
Sr	624	5.15	1.212	0.996
Co	873	8.44	1.317	0.999
Cd	1528	11.60	0.907	0.999
Zn	747	7.09	1.060	0.998
Cu	17,478	45.81	1.032	0.999
Pb	85,639	216.77	1.002	0.999
